# Hypoalbuminemia in HIV-infected patients: its determinants and correlation with CD4 count in Northern Uganda

**DOI:** 10.1186/s12981-025-00757-1

**Published:** 2025-09-02

**Authors:** Abukar Ali Ahmed, Hanan Asad Hassan, Venance Emmanuel Mswelo, Awil Abdulkadir Abdi, Onyanga Nixson, Hanaa Mohamed Shiekh Omar, Mohamed Jayte, Mohamed Elmalik Musa, Abishir Mohamud Hirsi

**Affiliations:** Department of Internal Medicine, Faculty of Clinical Medicine and Dentistry, Kampala International University, Ishaka, Bushenyi, Uganda

**Keywords:** Hypoalbuminemia, AIDS, HIV, CD4 cell count, Correlation, ART, Viral load

## Abstract

**Introduction:**

Hypoalbuminemia is linked to an earlier onset of acquired immune deficiency syndrome and increased mortality in patients living with HIV infection. Serum albumin is therefore an independent factor for the prediction of disease progression and mortality in People Living With HIV.

**Methods:**

This was a cross-sectional study conducted at Lira Regional Referral Hospital in northern Uganda that targeted HIV-positive outpatients attending the ART clinic with a sample size of 373 patients. Data were collected through structured interviews and laboratory tests in which the serum albumin concentration, viral load, and CD4 count were measured.

**Results:**

The prevalence of hypoalbuminemia was 19.6% (73/373). A moderate positive correlation was observed between the serum albumin concentration and the CD4 count (rs = 0.43, *p* < 0.001). Patients with no formal education [AOR = 2.03, 95%CI = 1.69–2.07, *P* = 0.03] were 2.03 times more likely to have hypoalbuminemia than those who had a tertiary/university education level. The odds of having hypoalbuminemia [AOR = 2.17, CI = 1.80–3.06, *P* = 0.02] were 2.17 higher among HIV-infected patients who were naïve ART than among those who were on ART. Additionally, the odds of having hypoalbuminemia [AOR = 2.91, CI = 2.13–3.66, *P* = 0.01] were 2.91 higher among HIV-infected patients who were in stage four than among those who were in stage 1.

**Conclusion:**

Hypoalbuminemia prevalence was high in PLWHIV, and a moderate positive correlation was found between the serum albumin level and the CD4 cell count. Lower education level, not being ART, and advanced HIV disease were independently associated with hypoalbuminemia.

**Supplementary Information:**

The online version contains supplementary material available at 10.1186/s12981-025-00757-1.

## Introduction

Recent studies have suggested the use of serum albumin level as a predictor marker of Human Immunodeficiency Virus disease severity and mortality in People Living With HIV [1]. A single measurement of albumin at baseline has been found to be useful in predicting survival of PLWHIV, especially those with CD4 counts < 200 cells [2]. Several factors contribute to the development of hypoalbuminemia in PLWHIV, such as increased albumin leakage during inflammatory response disease due to chronic inflammation, increased transcapillary escape of albumin due to impaired barrier function of the endothelium caused by the HIV-1 Tat1 protein, HIV infection-induced liver damage, diminished microbial clearance, impaired or decreased protein synthesis that enhances inflammation and reduced food intake due to decreased appetite [3–5]. Albumin can also be lost through urine (microalbuminuria) due to HIV-associated nephropathy in PLWHIV [6]. Serum albumin concentrations less than 3.5 g/dL are referred to as hypoalbuminemia [7]. The prevalence of hypoalbuminemia in HIV-positive individuals ranges from 5.9–39.5%^5^.

This condition has significant clinical implications, as it has been notably linked to earlier onset of acquired immune deficiency syndrome and increased mortality [1, 8]. A study found that for every 2.5 mg/L decrease in the serum albumin concentration, there is a corresponding 16% and 39% increase in the likelihood of an extended hospital stay and an increased chance of mortality, respectively, in critically ill inpatients, including patients with HIV/AIDS [9]. The risk of mortality in PLWHIV is 4.52 times higher for people with hypoalbuminemia than for those with serum albumin values ≥ 3.5 g/L^10^. Additionally, the risk of mortality in PLWHIV decreased by 6% with every 1 g/L increase in the serum albumin concentration [11]. Several studies revealed a positive correlation between the serum albumin concentration and the CD4 count [12–14]. Furthermore, studies reported that serum albumin is a useful prognostic marker for HIV/AIDS care and independent predictor marker of non-AIDS morbidity in PLWH, such as cardiovascular diseases [8, 15, 16]. While there is a dearth of knowledge regarding hypoalbuminemia and its utilization as a prognostic marker in HIV/AIDS care in Uganda. Our study provides the first comprehensive evaluation of hypoalbuminemia in PLWHIV in northern Uganda. A better understanding of hypoalbuminemia and its immune-virological correlations and associated factors can aid in identifying high-risk populations and tailoring interventions accordingly [13, 16–19]. This study seeks to determine the correlation between serum albumin and CD4 cell count, and identify the determinants of hypoalbuminemia in PLWHIV.

## Methodology

### Study design

This study was a cross-sectional study.

### Study setting

The study was carried out in Northern Uganda’s Lira district at the Lira Regional Referral Hospital. LRRH has ART clinics and provides antiretroviral therapy (ART) services, including care through units with integrated HIV services and community drug distribution. Currently, there are more than 12,000 HIV/AIDS clients receiving HAART [20].

### Inclusion criteria

The study included all HIV-positive patients aged 18 years and above attending the ART Clinic at Lira Regional Referral Hospital and who provided consent to participate in the study.

### Exclusion criteria

All patients living with HIV who met the inclusion criteria but were severely ill and had known liver disease or kidney disease were excluded from the study.

### Sample size estimation

Leslie Kish’s formula was used to determine the sample size of 373 participants for this study.

Leslie Kish’s formula for determining sample size in a finite population was used:

$$\:n=\frac{{n}_{0}}{1+\frac{{n}_{0}-1}{N}}$$where:

n = final sample size adjusted for finite.

population.

n_0_ = initial sample size (for an infinite.

population)

N = total population size.

where:

- n_0_ is the sample size for an infinite population, calculated as:

$${n_0} = \frac{{{z^2}P(1 - P)}}{{{d^2}}}$$

Where.

• n_0_ is the sample size for an infinite population.

• Z is the Z-score corresponding to the confidence level (typically 1.96 for 95% confidence).

• P is the assumed proportion of the population (50% or 0.5).

• d is the margin of error (commonly 5% or 0.05).

The sample size for this study was determined by calculating the predicted prevalence of 50%, as no recent study had been conducted in the local regions. The value utilized for P in the calculation was 0.5.

The required sample size for a finite population of 12,000, using Leslie Kish’s formula with a 50% population proportion and a 5% margin of error, is approximately **373** respondents.

### Sampling procedure

The PI employed a systematic random sampling technique for this study. Lira Regional Referral Hospital has multiple ART clinics for outpatients. On average, 1000 patients visit the clinic monthly to seek service. To get the sampling interval, the PI divided 373 study responders by 3000(average patients visit 3 in months), giving about 8 persons. This implies that the PI selected the study participants at an interval of three (every 8th patient with HIV voluntarily participated in the study daily). The first study participant was randomly selected from this, then followed participants were selected by adding the sampling interval 8 to the previous selection, continuing this process until all actual participants (373) were obtained.

### Recruitment of research assistants

The research assistants included 1 nurse, 1 clinical officer, and 2 lab technicians working at the LRRH ART clinic. Their role was to assist the principal investigator (PI). The nurses also obtained blood samples from the participants. The role of the lab technicians was to facilitate blood sample collection and transport those samples to the laboratory safely and appropriately for analysis at the main laboratory. A full day of training was provided to the research assistants, covering research methodology, data collection techniques and instruments, ethics, and code of conduct.

### Study procedure

The principal investigator and his assistant screened HIV-positive patients from the LRRH-ART clinic. The participants were chosen at random, all eligible participants were provided additional information about the research project, including risks and benefits. The participants were asked to sign a written informed consent document indicating their willingness to participate. After obtaining the informed consent focused history and physical examination were performed by the principal investigator (PI), and the findings were recorded on a pretested questionnaire. Blood sample (10 ml) were drawn from each participant’s median cubital vein and distributed as follows: 4 ml into EDTA Tube for HIV VL, 3 ml into a red top tube for serum albumin, and 3 ml into a purple top for CD4 cell count). The samples were sent to both the ART clinic laboratory and the main central laboratory LRRH. CD4 cell count was measured using the PIMA™ analyzer, and serum albumin was measured using the Coba311 biochemistry analyzer, and HIV VL was sent to the main central laboratory of LRRH.

## Study variables

### Independent variables

**Sociodemographic factors such as** age, sex, area of residence, marital status, level of education, occupation, and level of income.

**Medical factors such as** WHO Staging of HIV, BMI, Duration of HIV infection, current duration on ART, drug adherence, CD4 cell count, and HIV Viral Load.

### Dependent variable

The dependent variable for this study was hypoalbuminemia among patients living with HIV.

### Quality control

The data collection tool was pretested for validity and reliability, and adjustments were made before data collection. Research assistants were trained on the study objectives and protocol. The principal investigator double-checked the data daily for accuracy. Quality control measures included regular calibration of laboratory equipment, to control the material, and random samples were sent to the main laboratory (Lira Regional Referral Hospital an officer took those samples to the Laboratory with appropriate transportation).

### Data management and analysis

Microsoft Excel was used for entering the data. This was input twice and checked for errors via validation. For the final analysis, the data was exported to STATA version 16. The data was saved in storage devices and kept in various safe locations. Descriptive statistics were used for prevalence determination and presented as percentage with 95% confidence intervals. Spearman’s rank correlation coefficient was used to determine the correlation between the serum albumin concentration and the CD4 count. Both bivariate and multivariable logistic regression were used to identify the factors associated with hypoalbuminemia. A factor was taken into consideration for multivariable analysis if a p value of less than 0.2 was obtained during bivariate analysis. Multivariate logistic regression analysis was used to evaluate confounding factors and interactions. Statistical significance was accorded to factors that presented p values of less than 5% during multivariable analysis.

### Study limitations

This study was a cross-sectional design limits causal inferences, and the single-center setting may restrict generalizability.

## Results

### Sociodemographic and clinical characteristics of the patients with HIV attending ART clinic LRRH

The study enrolled 373 HIV-positive patients from the ART clinic Lira Regional Referral Hospital during the study period between the 1st October and 31st December 2024.

As summarized in Table 1, the majority of patients were male, 54.7% (204/373), and nearly one-third, 32.7% (122/373), had no formal education. Most of the patients (71.0%, 265/373) were on ART, and 45.6% (170/373) were classified as WHO HIV stage 1.

**Table 1:** Sociodemographic and clinical characteristics of HIV-infected patients attending the ART clinic at Lira regional referral Hospital(*N* = 373)

Variables	Frequencies (*N* = 373)	Percentage (%)
**Age categories (years)**		
< 30	96	25.7
31–40	105	28.2
41–50	93	24.9
> 50	79	21.2
**Sex**		
Male	204	54.7
Female	169	45.3
**Occupation**		
Employed	121	32.4
Unemployed	252	67.6
**Education level**		
Tertiary/University	68	18.2
Secondary	103	27.7
Primary	80	21.4
No formal education	122	32.7
**Marital status**		
Married	150	40.2
Single	126	33.8
Divorced/widowed	97	26.0
**BMI** kg/m2		
< 18.5 (underweight)	35	9.4
18.5–24.9 (normal)	197	52.8
25-29.9(overweight)	93	24.9
≥ 30(obese)	48	12.9
**Place of residence**		
Urban	219	58.7
Rural	154	41.3
**Average monthly income (Ugandan shilling** ***UGX*** **)**		
< 500,000	291	78.0
> 500,000	82	22.0
**Duration with HIV**		
< 5 years	124	33.2
5–10 years	118	31.6
> 10years	131	35.2
**Currently on ART**		
Yes	265	71.0
No	108	29.0
**Drug adherence (265)**		
High	179	67.5
Medium	69	26
Low	17	6.5
**WHO HIV stage**		
Stage I	170	45.6
Stage II	127	34.0
Stage III	43	11.5
Stage IV	33	8.8
**Viral load (copies /ml)**		
> 1000 copies	26	7.0
200–1000 copies	91	24.4
< 200 copies	256	68.6
**CD4 count (cells/mm** ^**3**^ **)**		
≥ 500	240	64.3
200–499	91	24.4
< 200	42	11.3

ART: Antiretroviral Therapy; BMI: Body Mass Index; HIV: Human Immunodeficiency Virus; UGX: Ugandan shillings; WHO: World Health Organization

### The prevalence of hypoalbuminemia among HIV-infected patients at LRRH

The overall prevalence of hypoalbuminemia in patients with HIV was 19.6% (73/373) (95%CI: 17.8–21.7%).

Correlation between hypoalbuminemia and CD4 count among HIV-infected patients.

A moderate positive correlation between serum albumin and CD4 cell count was observed, with a Spearman’s rank correlation coefficient of 0.43 and it was a statistically significant p value of < 0.001.

### Determinants of hypoalbuminemia among patients with HIV attending ART clinic LRRH

The determinants of hypoalbuminemia were analyzed using both bivariate and multivariate logistic regression models. At the bivariate logistic regression level, variables with a p-value < 0.2 were entered into the multivariable model for further analysis. The results of these analyses are presented in (Table 2).

The results from the multivariate logistic regression model in (Table 2) indicate that patients with no formal of education [AOR = 2.03, 95%CI = 1.69–2.07, *P* = 0.03] had higher odds of hypoalbuminemia than those who had tertiary/university education level. Patients who were not on ART had higher odds of Hypoalbuminemia [AOR = 2.17, 95%CI = 1.80–3.06, *P* = 0.02] than those who were on ART. Additionally, WHO HIV stage IV was associated with greater odds of hypoalbuminemia [AOR = 2.91, CI = 2.13–3.66, *P* = 0.01].

**Table 2:** Bivariate and multivariate logistic regression analyses of factors associated with hypoalbuminemia among HIV-infected patients attending Lira regional referral Hospital(*N* = 373)

Variable	Yes* 73(19.6%)		COR(95% CI)	*P* value	AOR(95% CI)	*P* value
**Age categories**						
< 30 years	16(21.9)		1.00		1.00	
30–40 years	23(31.5)		1.41(0.43–2.42)	0.18	1.63(0.21–3.56)	0.38
41–50 years	22(30.1)		1.54(0.31–1.97)	0.24	1.79(0.38–2.26)	0.29
> 50years	12(16.4)		0.89(0.06–0.95)	0.02	1.96(0.87–2.77)	0.84
**Education level**						
Tertiary/University	13(17.8)		1.00		1.00	
Secondary	10(13.7)		0.45(0.31–2.37)	0.31	0.39(0.65–2.81)	0.11
Primary	21(28.8)		0.88(0.54–1.95)	0.24	0.08(0.41–1.40)	0.15
No formal education	29(39.7)		2.41(1.54–3.82)	0.02	2.03(1.69–2.07)	0.03*
**Marital status**						
Married	24(32.9)		1.00			
Single	33(45.2)		1.85(1.21–3.95)	0.03	1.27(0.88–1.72)	0.07
Divorced/widowed	16(21.9)		0.76(0.22–1.97)	0.63	0.58(0.18–1.87)	0.49
**Monthly income (UGX)**						
< 500,000	51(69.9)					
> 500,000	22(30.1)		1.72(1.03–2.95)	0.04	1.41(0.27–3.93)	0.21
**Patient on ART**						
Yes	56(76.7)		1.00		1.00	
No	17(23.3)		15.71(5.11–17.4)	< 0.001	2.17(1.80–3.06)	0.02*
**Duration with HIV/AIDS**						
< 5 years	23(31.5)		1.00		1.00	
5–10 years	15(20.5)		0.64(0.29–2.75)	0.21	1.93(0.25–2.66)	0.29
> 10years	35(47.9)		1.60 (1.02–2.94)	0.04	1.10(0.92–2.75)	0.18
**WHO HIV staging**						
Stage I	41(56.2)		1.00		1.00	
Stage II	15(20.5)		1.18(0.13–3.66)	0.19	1.34(0.81–1.97)	0.18
Stage III	12(16.4)		2.92(0.21–2.15)	0.71	0.99(0.18–2.11)	0.37
Stage IV	5(6.8)		9.13(4.06–10.70)	0.006	2.91(2.13–3.66)	0.01*
**Viral load copies/ml**						
> 1000		19(26.0)	1.00		1.00	
200–1000		28(38.4)	0.16(0.05–3.26)	0.14	0.41(0.15–2.26)	0.14
< 1000		26(35.6)	0.04(0.01–0.98)	0.03	0.55(0.26–1.08)	0.07
**Drug adherence**						
High		30(41.1)			1.00	
Medium		34(46.6)	4.8(2.1–6.26)	0.31	5.29(0.76–9.76)	0.09
Low		9(12.3)	5.6(3.02–8.30)	< 0.001	4.65(0.47–12.66)	0.17

ART: Antiretroviral Therapy; AOR: adjusted odds ratio; COR: crude odds ratio; 95% CI: 95% confidence interval; HIV: Human Immunodeficiency Virus; P-value < 0.05 were considered statistically significant; UGX: Ugandan shillings; WHO: World Health Organization; Yes: patients with hypoalbuminemia;

## Discussion

The prevalence of hypoalbuminemia among HIV-infected patients attending Lira Regional Referral Hospital (LRRH) was found to be high at 19.6% (73/373). This finding highlights the importance of hypoalbuminemia as a common biochemical abnormality in HIV-infected individuals. The findings of our current study are slightly greater than those of a cross-sectional study conducted in Brazil, which reported a hypoalbuminemia prevalence of 11.7% among HIV-positive outpatients [5].

However, the prevalence is lower than that reported in a study conducted in India, where 66.3% of the subjects had hypoalbuminemia [14]. However, it is important to note that in the Indian study, most participants had a CD4 count of less than 350 cells/mm^3^, whereas in the present study, 64.3% of the participants had a CD4 count of more than 500 cells/mm^3^. Similarly higher prevalence of hypoalbuminemia was also reported in other studies conducted in India [1, 2, 13, 16–19].

A prospective cohort study conducted in Tanzania revealed that 39.5% of HIV infected patients had hypoalbuminemia at baseline [10]. This prevalence is slightly higher than that obtained in the present study. The Tanzania study was a prospective cohort study with a larger sample size. Moreover, in the Tanzanian study, serum albumin was measured at ART initiation, whereas in our present study, serum albumin was measured in all participants, regardless of ART status.

The current study revealed a moderate positive correlation between the serum albumin level and CD4 count among HIV-infected patients, with a statistically significant p value (*p* < 0.001). This finding indicates that as the serum albumin level increases, the CD4 count tends to increase, and the opposite is true of low serum albumin, which is associated with a lower CD4 count. The findings of our current study agree with those of a study by Balgi V et al.(2019), which also revealed a strong correlation between the serum albumin concentration and the CD4 count, with a statistically significant p value of < 0.003^14^. Similarly, a study conducted in India reported a strong positive correlation between the CD4 count and the serum albumin concentration, which was statistically significant *P* < 0.0001^18^. Both studies support our findings. Therefore, a low CD4 count is associated with decreased or reduced serum albumin levels, both of which indicate advanced disease. Additionally, other studies revealed an association between the serum albumin concentration and the CD4 count which are similar findings to our current study [13, 16].

Conversely, a cross-sectional study conducted in Cameroon reported a weak negative correlation between the CD4 count and total protein [21].

The findings of the present study highlight that hypoalbuminemia is associated with advanced HIV disease, and lower CD4 counts and high viral load counts were observed in patients with hypoalbuminemia. Therefore, these findings suggest that the serum albumin concentration could be a useful biomarker for monitoring immune status and disease progression in HIV-infected patients. Additionally, since serum albumin testing is inexpensive and widely available, it could be beneficial and particularly useful in resource-limited settings.

Patients who were not on ART had greater odds of having hypoalbuminemia than those on ART drugs. Similarly, Leal et al. reported that patients who were not exposed to ART were more likely to have hypoalbuminemia [5]. The findings of the present study highlight that ART initiation or being on ART is crucial in patients with HIV. Several studies highlighted changes or increased plasma albumin concentrations after initiation of Highly Active Antiretroviral Therapy (HAART) in patients living with HIV [22]. ART in HIV patients leads to viral load suppression and improved immune function, restoring the CD4 count [23].

The current study also revealed that patients with no formal education had greater odds of hypoalbuminemia than did those with tertiary/university education. However, these findings shed light on the need for longitudinal studies to assess the link between low serum albumin and low educational levels in patients with HIV. Education is closely linked to health literacy, access to healthcare, and the ability to make informed decisions about nutrition and treatment adherence.

The present study revealed that patients in WHO HIV stage IV were nearly three times more likely to have hypoalbuminemia than were those in stage I. Similar findings were reported that participants with WHO HIV stage IV had more odds of hypoalbuminemia than those in stage I [5, 10]. Advanced HIV disease leads to systemic inflammation and increased rates of opportunistic infection, which contributes to hypoalbuminemia in patients with HIV [24]. Studies have shown that the serum albumin concentration is a predictor of the progression of HIV-related advanced disease and AIDS-related mortality [5, 11].

## Conclusions

This study identified a high prevalence of hypoalbuminemia (19.6%) among PLWHIV at Lira Regional Referral Hospital, Northern Uganda, with a higher risk observed among patients who were not on ART, those with no formal education, and those with WHO HIV stage IV. These findings underscore the potential benefits of routine serum albumin screening in PLWHIV. Therefore, this study suggests that serum albumin, being inexpensive and widely accessible, could serve as a valuable prognostic tool for monitoring immune status and disease progression, particularly in resource-limited settings. However, the cross-sectional design limits causal inferences, and the single-center setting may restrict generalizability. Further multi-center longitudinal studies are recommended to explore causal relationships, disease progression, and short-term mortality in PLWHIV to validate these findings and inform broader public health strategies.

## Electronic supplementary material

Below is the link to the electronic supplementary material.

Supplementary Material 1

Supplementary Material 2

Supplementary Material 3

## Data Availability

Data is available on request by contacting corresponding author. Abukar Ali Ahmedahmed.abukar@studwc.kiu.ac.ugabukr252@gmail.com.

## Abbreviations

AIDs: Acquired immunodeficiency syndrome
ART: Anti-retroviral treatment
HAART: Highly Active Antiretroviral Therapy
HIV: Human immunodeficiency virus
LRRH: Lira regional referral hospital
PI: principal investigator
PLWHIV: People living with HIV
WHO: World Health Organization
