# Supplementary material for: Hypoalbuminemia in HIV-infected patients: its determinants and correlation with CD4 count in Northern Uganda
Source: AIDS Res Ther. 2025 Sep 2;22:88. doi: 10.1186/s12981-025-00757-1 (PMC12406449; doi:10.1186/s12981-025-00757-1)
Supplement: Supplementary file 1 — Supplementary Material 1 [file 12981_2025_757_MOESM1_ESM.docx]

I am writing to formally request a 100% waiver for the publication fees associated with my manuscript, " HYPOALBUMINEMIA IN HIV-INFECTED PATIENTS: ITS DETERMINANTS AND CORRELATION WITH CD4 COUNT IN NORTHERN UGANDA.

I am originally from Somalia and currently reside in Uganda, where I current studying master of Medicine. Due to my current financial situation and the economic conditions in Somalia and Uganda, I cannot afford the publication fees.

This research was conducted without external funding. Despite those challenges, I pursued this research due to its critical importance in addressing cardiovascular health issues in low-resource settings. I am committed to advancing knowledge in this field.

My research has significant implications for cardiovascular health, especially in low-resource settings, and I am eager to share my findings with the scientific community through your esteemed journal. A waiver would greatly assist me in this endeavor.
