# Supplementary material for: Hypoalbuminemia in HIV-infected patients: its determinants and correlation with CD4 count in Northern Uganda
Source: AIDS Res Ther. 2025 Sep 2;22:88. doi: 10.1186/s12981-025-00757-1 (PMC12406449; doi:10.1186/s12981-025-00757-1)
Supplement: Supplementary file 2 — Supplementary Material 2 [file 12981_2025_757_MOESM2_ESM.pdf]

TELEPHONES:

General lines 0473420023
Hospital Director 0372280238
FAX 0473420139
E-Mail: lirarrh@gmail.com
In any correspondence on this
Subject please quote Ref. No:

THE REPUBLIC OF UGANDA

MINISTRY OF HEALTH
LIRA REGIONAL REFERRAL
HOSPITAL
P.O. BOX 2, LIRA

29th October, 2024

The Principle
Kampala International University

Dear Sir/ Madam,

ABUKAR ALI AHMED – **Master of Medicine**

The above student from your institution has requested to carry out data collection titled: **"Hypoalbuminemia in HIV- Infected Patients: its determinant and correlation with CD4 Count" at** Lira Regional Referral Hospital"

This letter is to confirm that she has been granted permission to carry out his research in Lira Regional Referral Hospital from **1st November 2024** to **31st January 2025** under the supervision of the **Officer in charge.**

Thank you.

FOR: HOSPITAL DIRECTOR
P.O BOX 2. LIRA
29 OCT 2024
LIRA REGIONAL REFERRAL HOSPITAL

pp

Mr. Otema Charles Lwanga
For: HOSPITAL DIRECTOR

cc: Head of Unit
cc: Human Resource Office – LRRH
cc: Principal Hospital Administrator
cc: File

Scanned with CamScanner
