# Supplementary material for: Hypoalbuminemia in HIV-infected patients: its determinants and correlation with CD4 count in Northern Uganda
Source: AIDS Res Ther. 2025 Sep 2;22:88. doi: 10.1186/s12981-025-00757-1 (PMC12406449; doi:10.1186/s12981-025-00757-1)
Supplement: Supplementary file 3 — Supplementary Material 3 [file 12981_2025_757_MOESM3_ESM.pdf]

KAMPALA INTERNATIONAL UNIVERSITY

Western Campus
P O BOX 71 Ishaka, Uganda
Tel: +256 758 096 775
Email: kiurec2017@kiu.ac.ug
Website: www.kiu.ac.ug

KIU RESEARCH ETHICS COMMITTEE(REC)
APPROVED
VALID UNTIL
11 SEP 2025
P.O. Box 71, Bushenyi - Ishaka

RESEARCH ETHICS COMMITTEE (REC)

11/09/2024

To: ABUKAR AHMED

KAMPALA INTERNATIONAL UNIVERSITY
+256708588759

Type: Initial Review

Re: KIU-2024-401: HYPOALBUMINEMIA IN HIV-INFECTED PATIENTS: ITS DETERMINANTS AND CORRELATION WITH CD4 COUNT AT LIRA REGIONAL REFERRAL HOSPITAL

I am pleased to inform you that at the **80** convened meeting on **14/08/2024**, the KIU REC meeting voted to approve the above referenced application.
Approval of the research is for the period of **11/09/2024** to **11/09/2025.**

As Principal Investigator of the research, you are responsible for fulfilling the following requirements of approval:

1. All co-investigators must be kept informed of the status of the research.
2. Changes, amendments, and addenda to the protocol or the consent form must be submitted to the REC for re-review and approval **prior** to the activation of the changes.
3. Reports of unanticipated problems involving risks to participants or any new information which could change the risk benefit: ratio must be submitted to the REC.
4. Only approved consent forms are to be used in the enrollment of participants. All consent forms signed by participants and/or witnesses should be retained on file. The REC may conduct audits of all study records, and consent documentation may be part of such audits.
5. Continuing review application must be submitted to the REC **eight weeks** prior to the expiration date of **11/09/2025** in order to continue the study beyond the approved period. Failure to submit a continuing review application in a timely fashion may result in suspension or termination of the study.
6. The REC application number assigned to the research should be cited in any correspondence with the REC of record.
7. You are required to register the research protocol with the Uganda National Council for Science and Technology (UNCST) for final clearance to undertake the study in Uganda.

The following is the list of all documents approved in this application by KIU REC:

Scanned with CamScanner

No.	Document Title	Language	Version Number	Version Date
1	REC-Compliance Report	English	2	2024-09-05
2	Protocol	English	2	2024-09-05
3	PLAGIARISM	English	1	2024-08-12
4	CVs of the investigators	English	1	2012-08-12
5	Letter department supervisor	English	1	2024-08-12
6	Letter department supervisor	English	1	2024-08-12
7	Informed Consent forms	Langi	1	2024-08-12
8	Informed Consent forms	English	1	2024-08-12
9	Data collection tools	English	1	2024-08-12
10	Data collection tools	Langi	1	2024-06-14

Yours Sincerely

KIU RESEARCH ETHICS COMMITTEE(REC)
APPROVED
VALID UNTIL
11 SEP 2025
P.O. Box 71, Bushenyi - Ishaka

Kiswezi Ahmed
For: KIU REC

Scanned with CamScanner
